# Continuous variable quantum optical simulation for time evolution of quantum harmonic oscillators

**DOI:** 10.1038/srep22914

**Published:** 2016-03-10

**Authors:** Xiaowei Deng, Shuhong Hao, Hong Guo, Changde Xie, Xiaolong Su

**Affiliations:** 1 State Key Laboratory of Quantum Optics and Quantum Optics Devices, Institute of Opto-Electronics, Shanxi University, Taiyuan 030006, China; 2 Collaborative Innovation Center of Extreme Optics, Shanxi University, Taiyuan 030006, China; 3 College of Physical Science and Technology, Central China Normal University, Wuhan 430079, China

## Abstract

Quantum simulation enables one to mimic the evolution of other quantum systems using a controllable quantum system. Quantum harmonic oscillator (QHO) is one of the most important model systems in quantum physics. To observe the transient dynamics of a QHO with high oscillation frequency directly is difficult. We experimentally simulate the transient behaviors of QHO in an open system during time evolution with an optical mode and a logical operation system of continuous variable quantum computation. The time evolution of an atomic ensemble in the collective spontaneous emission is analytically simulated by mapping the atomic ensemble onto a QHO. The measured fidelity, which is used for quantifying the quality of the simulation, is higher than its classical limit. The presented simulation scheme provides a new tool for studying the dynamic behaviors of QHO.

The simulation for dynamic behaviors of a quantum system in general is difficult with any classical configurations. In 1982, R. Feynman envisaged a quantum simulator, which should be built of quantum mechanical elements, and thus it can naturally evolve according to quantum mechanics laws^1^. By using quantum simulation (QS), we can simulate the dynamic evolution of other quantum system with a controllable quantum system^2^,^3^,^4^,^5^. In recent years the study on QS has been growing rapidly since the development of quantum information science has prepared enough technologies to implement the manipulation of quantum systems and realize QS. Moreover, many potential applications of QS in variety of scientific fields are a strong motivation to push it progressing. Sizable theoretical proposals on QS have been proposed in the past decade^5^. In the experimental investigations the ultra-cold quantum gases^6^, trapped ions^7^,^8^, photonic system^9^,^10^, super-conducting circuits^11^, nuclear magnetic resonance (NMR)^12^,^13^,^14^,^15^,^16^, nitrogen-vacancy centers^17^, and so on, have been used as the controllable quantum systems to implement QS. Some experiments on simulating the dynamic processes in the condensed-matter, chemical, biological and frustrated systems have also been demonstrated^5^,^18^,^19^,^20^. In the achieved QS experiments, the used quantum variables are discrete variables (DV) with finite dimensions. Usually, high fidelity can be obtained in the DVQS system, such as trapped ions^7^, photonic^10^, and NMR^13^,^14^,^15^ systems.

Although the continuous variable (CV) quantum information and quantum computation (QC) based on applying quadrature amplitude and phase of quantized optical mode (qu-mode) to be quantum variables have been extensively explored^21^,^22^,^23^,^24^,^25^,^26^,^27^,^28^,^29^, QS scheme utilizing qu-mode and logical operations in CVQC to mimic the dynamics of the other quantum systems has not been implemented before. Very recently, a QS scheme of quantum field theory using continuous variables is proposed^30^.

In physics the dynamics of numerous systems is governed by the harmonic oscillator equations, thus the study for dynamic behaviors of harmonic oscillators has general significance. As an important model system in quantum mechanics, quantum harmonic oscillator (QHO) has been extensively investigated. However, it is difficult to observe the transient behaviors of the quantum state of a high frequency QHO in the time evolution directly. Untill now, only the truncated QHO was simulated with NMR system in DV regime^12^. As well-known qu-mode is a typical QHO, which can be applied to simulate the dynamics of some other QHO systems. For example, the direct experimental observation of the dynamics of an atomic ensemble in the collective spontaneous emission is difficult, but it can be simulated by the time evolution of a QHO^31^.

In this paper, we present the first CV quantum optical simulation (CVQOS) experiment, in which a qu-mode and a logical operation unit used in CVQC serve as the controllable quantum system to simulate the dynamic behavior of an other quantum system. At first, the transient behavior of a QHO in an open system is experimentally recorded and then the dynamics of an atomic ensemble in the collective spontaneous emission is analytically simulated by mapping an atomic ensemble onto a QHO as an example. The presented CVQOS enables one to simulate the time evolution of the quantum state in collective spontaneous emission of an atomic ensemble. Under the condition of satisfying the limitations imposed by the Holstein-Primakoff transformation (HPT)^31^, the time evolution of the uncertainty of the collective spin component, the spin-squeezing parameter and the population of atomic upper-state during the collective spontaneous emission are analytically mimicked by the CVQOS. The fidelity of the presented simulation is higher than the corresponding classical limit. If more qu-modes and logical operations are applied the presented scheme perhaps can be extended to simulate a multimode system, such as the evolution of coupled harmonics oscillators^32^,^33^.

## Results

### Time evolution of a quantum harmonic oscillator

The Hamiltonian of a QHO is 
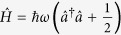
, in which *ħ* is the Plank constant, *ω* is the resonant frequency of the oscillator, 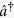 and 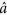 are the creation and annihilation operators, respectively. In an open system, the QHO will interact with the environment and under the rotating wave approximation the Hamiltonian is given by 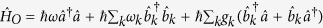, where the second and third terms represent the environment and the interaction between the QHO and environment, respectively, *g*_*k*_ is the interaction coefficient, 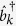 and 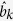 are the creation and annihilation operators of the k-th mode in the vacuum reservoir, respectively. According to the Hamiltonian of a QHO in an open system with vacuum environment, the time evolution of the dimensionless position and momentum operators 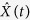 and 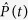 are expressed by


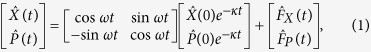


where 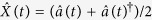 and 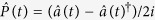, *κ* is the decay rate, and 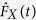 and 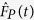 are the noise operators depending on the reservoir variables, which guarantees the time-invariant of the commutation relation between position and momentum operators^34^. For the vacuum reservoir we have 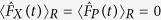, 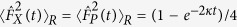, and 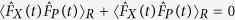, where the subscript *R* represents the average of the reservoir. When *κ* = 0, there is no interaction between the QHO and the vacuum reservoir, which means that the QHO is in a closed system. When 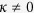, Eq. (1) describes the case in an open system, where 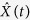 and 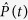 will attenuate with time. From Eq. (1) we can see, if taking *θ* = *ωt*, the time evolution of a QHO can be simulated by a rotation operation of a qu-mode in phase space^24^. In this way, we can observe the real-time evolution of the quantum state of a high frequency QHO and record the transient process of the rapid evolution easily.

**Figure 1:**
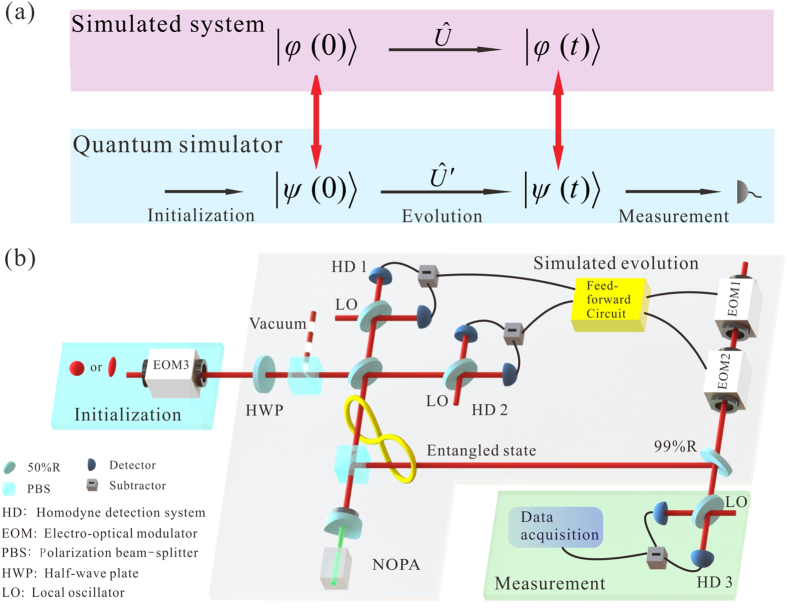
Schematic and experimental set-up of the quantum simulation system. (**a**) The simulated quantum system evolves with the unitary of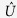, the quantum simulator consisting of three parts: initialization, simulated evolution with the unitary of 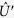 and measurement. (**b**) The experimental set-up. The part of initialization is for the preparation of input state. In the simulated evolution, the input state after the interaction with the environment and a submode of the EPR entangled state are coupled on a 50% beam-splitter, and then the output beams are measured by the homodyne detection systems (HD1 and HD2) with measurement angles *θ*_1_ and *θ*_2_, respectively. The measurement results of them are combined together in the feedforward circuit, and are fedforward to the other submode of the EPR entangled state through EOM1, EOM2 and a 99% reflection beam-splitter. Finally, in the part of measurement, the output state is measured by the third homodyne detection system HD3.

A schematic for quantum simulation is shown in Fig. 1(a), The simulated system evolves from the initial state 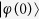 to 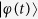, where 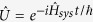, and 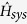 is the Hamiltonian of the simulated system. The quantum simulator is a controllable system consisting of three parts: preparing the initial state 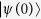 (initialization), implementing the desired unitary evolution 
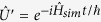
 (evolution), and measuring the final state 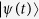 (measurement)^5^. If a mapping between the simulated system and the simulator 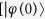 and 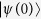, 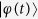 and 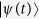, 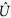 and 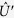] exists, the system can be simulated by the quantum simulator.

The experiment set-up is shown in Fig. 1(b). In the experiment, the qu-modes in a coherent and an amplitude-squeezed state with −3.5 dB squeezing are used as input states to simulate the time evolution of other QHOs, respectively. The amplitude of the coherent (squeezed) state is 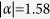, which is obtained by applying a 10 dB amplitude modulation at 2 MHz on the input state by means of an electro-optical modulator (EOM3). The rotation operation is implemented by means of an ancillary Einstein-Podolsky-Rosen (EPR) entangled state with −3.5 dB squeezing (i.e. the squeezing parameter *r*_*e*_ = 0.40), which is generated from a non-degenerate optical parametric amplifier (NOPA)^35^,^36^,^37^. A continuous wave intracavity frequency-doubled and frequency-stabilized Nd:YAP/LBO (Nd-doped YAlO3 perorskite/lithium triborate) laser is used to pump the NOPA. The NOPA consists of an *α*-cut type-II KTP crystal and a concave mirror. The front face of the KTP was coated to be used for the input coupler and the concave mirror serves as the output coupler of the entangled state. The transmittance of the input coupler at 540 nm and 1080 nm are 99.8% and 0.04%, respectively. The transmittance of the output coupler at 540 nm and 1080 nm are 0.5% and 5.2%, respectively. The NOPA is operated at de-amplification condition, which corresponds to lock the relative phase between the pump laser and the injected signal to (2*n* + 1)*π* (*n* is the integer). In this way, the EPR entangled state with anti-correlated amplitude and correlated phase quadratures is generated.

Another NOPA is used to generate the amplitude-squeezed state, which is also used as an input state. When the NOPA is operated at de-amplification condition, the coupled-modes of two EPR entangled beams at +45° and −45° polarization directions are the quadrature-amplitude and the quadrature-phase squeezed states, respectively^38^. The measured squeezing and anti-squeezing noises of the quadrature-amplitude squeezed state are −3.5 dB and 8.5 dB, respectively. The quantum efficiency of the photodiode (FD500W-1064, Fermionics) used in the homodyne detection system is 95%. The interference efficiency on a beam-splitter is about 99%. The fluctuation of the phase locking system is about 2–3°.

In the experiment, a CV teleportation based scheme is used to implement the rotation operation of a qu-mode. An input mode is coupled to a submode of the EPR entangled state via a 50% beam-splitter. The two output modes of the beam-splitter are measured by two homodyne detection systems HD1 and HD2 with measurement angles *θ*_1_ and *θ*_2_, respectively. The measured results are combined together in the feedforward circuit and then are fedforward to the other submode of the EPR entangled state by EOM1, EOM2 and a 99% reflection beam-splitter. The specific features of such a rotation operation scheme are: 1. the output quantum state is not destroyed and its quantum features can be retained. 2. Since, in fact, the rotation operation based on CV teleportation is a logical gate in CV quantum computation^21^,^22^,^23^,^24^,^25^,^26^,^27^,^28^,^29^ and thus it can be integrated in a gate sequence to simulate a complex quantum dynamics.

In the teleportation based rotation operation scheme, the measurement angles *θ*_1_ and *θ*_2_ in HD1 and HD2 are controlled to simulate the evolution of the input state. The measurement results of HD1 and HD2 are 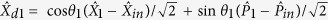 and 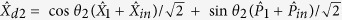, where 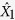 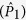 and 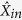 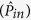 are the amplitude (phase) quadrature of one EPR entangled mode and input mode, respectively. The output state of a qu-mode in a closed system is expressed by


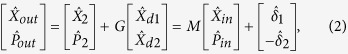


where 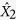 and 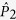 are amplitude and phase quadratures of the other EPR entangled mode, and


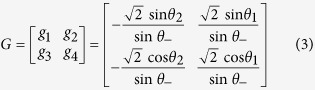


is the gain factor in the feedforward loop, in which *θ*_+_ = *θ*_1_ + *θ*_2_ and *θ*_−_ = *θ*_1_ − *θ*_2_, respectively. 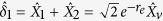 and 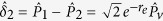 are the excess noise of the EPR state, where 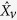 and 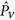 represent the amplitude and phase quadratures of a vacuum state. Obviously, in the ideal case with infinite squeezing (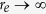), these excess noises will vanish, and the better the squeezing is, the smaller the noise terms are. When *θ*_1_ = *θ*_2_ +3*π*/2, *θ*_2_ = θ/2, the transformation matrix


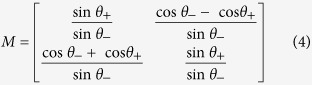


becomes the rotation transformation matrix 
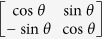
, and the dynamic behavior of the QHO at *ωt* = *θ* will be exhibited.

In the experiment process, we calculate the gain factor and transformation matrixes *G* and *M* according to equations (3) and (4) for a certain rotation angle *θ* at first. Then, the measured results 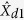 and 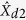 of HD1 and HD2 are combined together according to 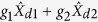 and 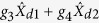 in the feedforward circuit, and are fedforward to the amplitude and phase quadratures of the other submode of the EPR entangled state, respectively. The output state is measured by the third homodyne detection system HD3. In HD3, the relative phase between the local oscillator and the output optical beam is scanned with a frequency of 2 Hz, which enables one to perform quantum tomography^39^,^40^. The AC output signal from HD3 is mixed with a local reference signal of 2 MHz, then it is filtered by a low-pass filter with a bandwidth of 10 kHz and amplified 1000 times (Low noise amplifier, SRS, SR560). Finally, it is recorded by a digital storage oscilloscope. The DC output from HD3 is used as a trigger signal to record the output results. The sampling rate is chosen to be 500 kpts/s. About 50000 data points are used to reconstruct the Wigner function of the output state.

Generally, the interaction between the input state and vacuum environment results in the linear attenuation of the amplitude of the qu-mode can be mimicked by an adjustable beam-splitter composed by a half wave plate (HWP) and a polarization beam-splitter (PBS). The rate of the attenuation is proportional to the strength of the interaction and the change of the interaction coefficient is mimicked by adjusting the transmittance of the beam-splitter. For an open system with the vacuum environment, the output state becomes


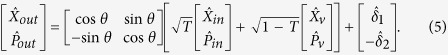


Comparing Eq. (5) with Eq. (1), we can see that the de-coherence of the input state induced by the environment is exactly simulated by the beam-splitter with 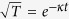.

### Experimental results

**Figure 2:**
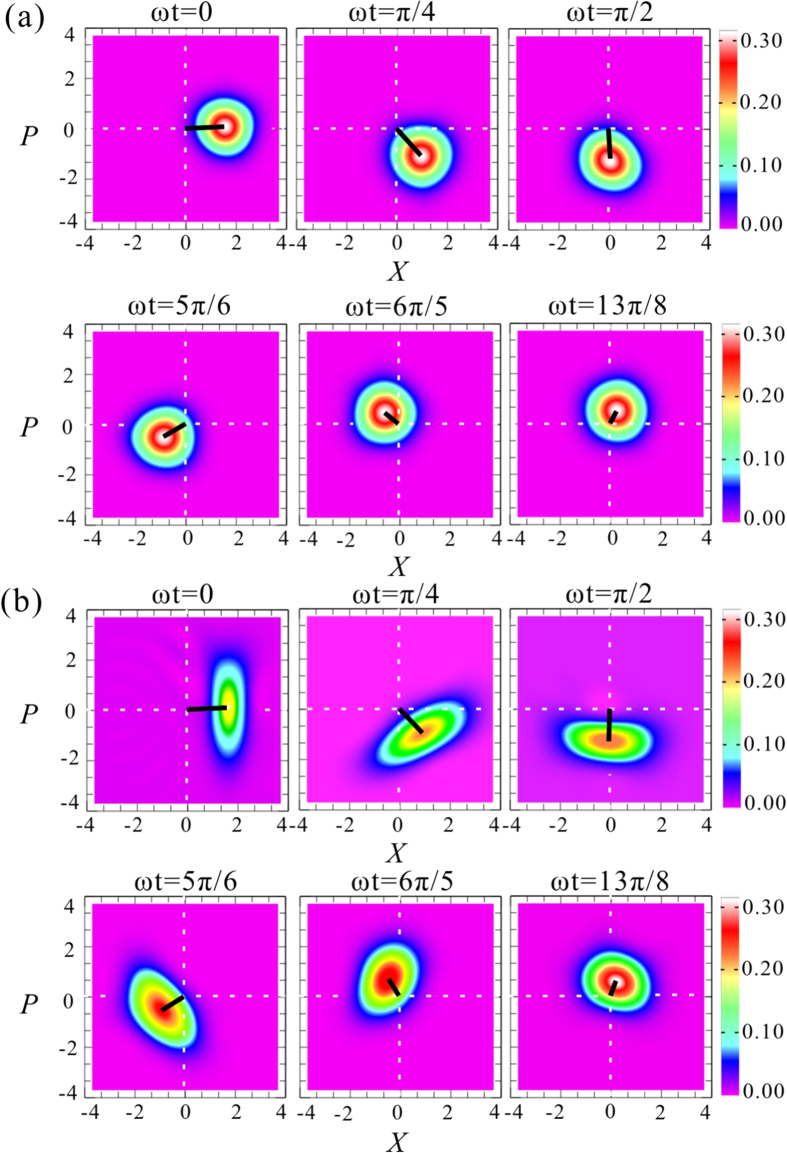
The time evolution of the quantum harmonic oscillator in phase space under the condition of de-coherence. (**a**,**b**) Are time evolution of the QHO with a coherent and a squeezed state as input state, respectively. The black lines represent amplitude of the quantum states.

When *T* < 1 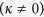, the system corresponds to the QHO in the open system. By adjusting the transmission coefficient T of the beam-splitter, the different damping is simulated. Figure 2 shows the time evolution of a QHO in an open system for an initial coherent state [Fig. 2(a)] and an initial amplitude-squeezed state [Fig. 2(b)], respectively, which are the top view of the measured Wigner function of the output state (See supplementary information for details) in phase space at different time points. The rotation of the quantum state of the input qu-mode in phase space and the gradually decreasing of the amplitude of the QHO (black lines) during the time evolution are exhibited. The measured amplitudes of the coherent (squeezed) states in phase space are 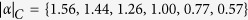 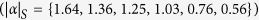 with 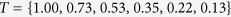 at 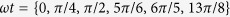, which correspond to the existence of a damping (the decay rate) with *κ*/*ω* = 0.2. In this case, during the evolution of the QHO the property of coherent state keeps unchanging only its amplitude decreases gradually, while the feature of the squeezed state is changed. The squeezing direction rotated along with the rotation of the quantum state. The squeezing degree of the squeezed state is decreased along with the attenuation of the amplitude and the state trends to a coherent state gradually, that just shows actively the de-coherence effect of a squeezed state in the open system.

At the same time, the maximum of the Wigner function increases gradually during the time evolution. The measured maximum of the Wigner function with an input coherent state is about 0.33. The maximum values of the Wigner functions for a pure squeezed state (at minimal-uncertainty state) are same with that of a coherent state. However, the squeezed state prepared experimentally is not a pure state, and thus its squeezing and anti-squeezing noises are different. The difference between the squeezing and anti-squeezing noises of the prepared squeezed state leads to the broadening of the Wigner function and the decrease of the maximum. In this case, the measured maximum of the Wigner function are {0.20, 0.21, 0.22, 0.24, 0.26, 0.29} at 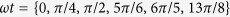, respectively.

### Simulation of collective spontaneous emission of an atomic ensemble

Based on HPT, the model of QHO has been extensively applied in theoretical and experimental study of atomic ensembles^41^,^42^. Collective spontaneous emission of an atomic ensemble induced by vacuum is one of the key cause resulting in the de-coherence of the atomic ensemble^43^. The Hamiltonian of an atomic ensemble consisting of *N* (*N* ≫ 1) identical two-level atoms interacting with a vacuum reservoir is expressed by 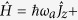

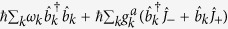
, where *ω*_*a*_ is the atomic transition frequency, 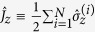 is z component of the collective spin operator of the atomic ensemble, 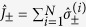 are the ladder operators of the collective spin and 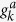 is the coupling coefficient between atoms and the *k*-th mode of the vacuum reservoir^44^. In what follows the state of an atomic ensemble we analyzed is within the Hilbert subspace of total spin angular momentum operator 
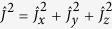
 with eigenvalue 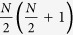, i.e. the permutation-symmetric subspace of the N-atom system, where 
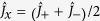
 and 
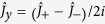
 are x and y component of the collective spin operator of an atomic ensemble, respectively. In the case of *N* ≫ 1 and 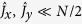, using HPT we have 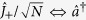, 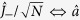, 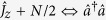, and thus the effective Hamiltonian can be written as 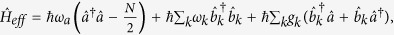 where 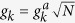
41. It is evident that the difference between 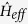 and 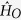 is only a constant 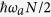, so the dynamics of an atomic ensemble in the collective spontaneous emission can be simulated by the presented CVQOS.

The relations between the collective spin operators of an atomic ensemble and the position (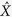) and momentum (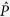) operators of a QHO obtained from HPT are given by 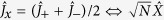, 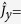 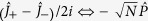, and 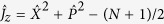. A spin coherent (squeezed) state of an atomic ensemble is mapped onto a coherent (squeezed) state of a QHO (qu-mode), respectively (See supplementary information for details).

**Figure 3:**
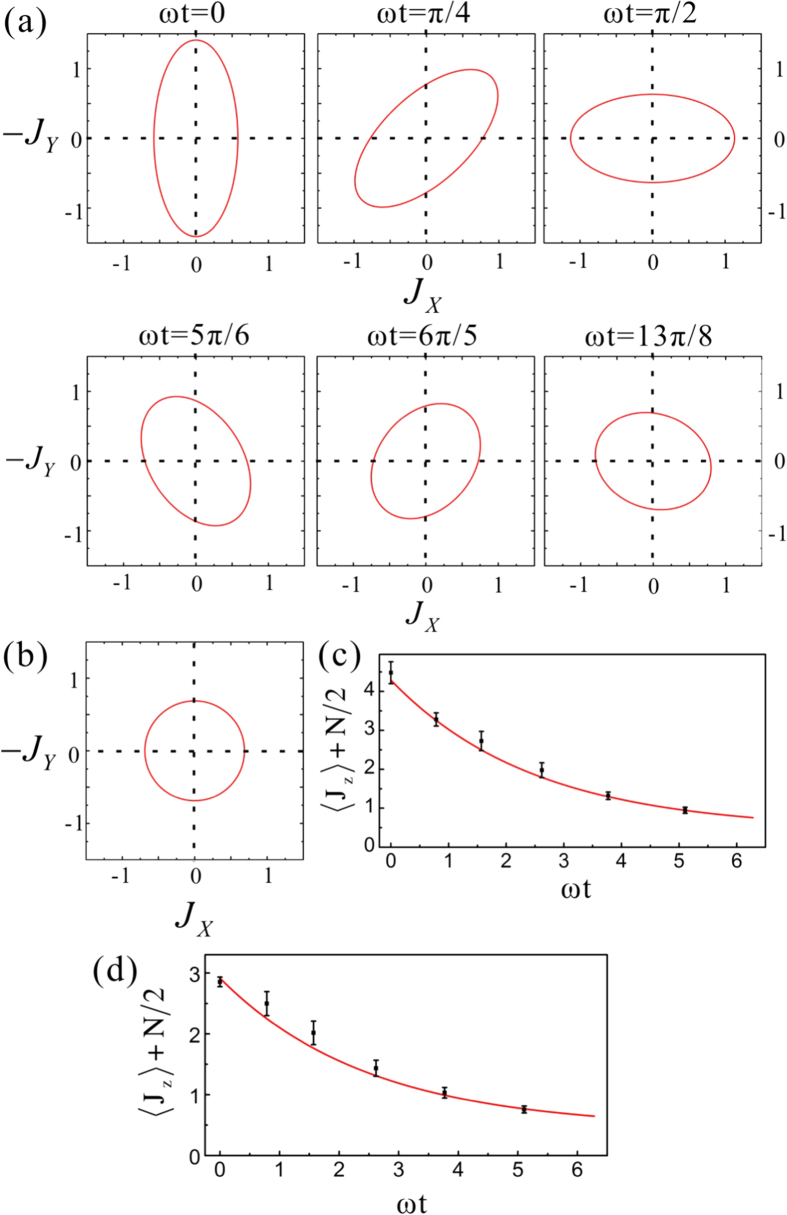
The dynamic behavior of the atomic ensemble in the collective spontaneous emission. (**a**,**b**) Are the uncertainty 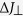 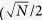 is used as an unit) for the initial spin squeezed and coherent states of an atomic ensemble at different time points, respectively. (**c**,**d**) Are the time evolution of total upper-state population for the initial spin squeezed and coherent states, respectively. The black dots correspond to the time points connected with in Fig. 2(a,b).

We analytically simulate the dynamics of an atomic ensemble in the collective spontaneous emission with the experimentally obtained parameters in the subsection of experimental results. The evolution of uncertainty 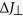 of the collective spin component 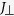 (See supplementary information for details), which is perpendicular to the collective spin average vector and shown in Fig. 3(a,b). Figure 3(a) shows the uncertainty 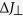 at different time points in the case where the spin squeezed state is the input state. Apparently, the direction of the minimal uncertainty 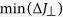 rotates around the z-axis with the angular rate 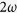. The spin-squeezing parameter 
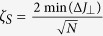
 increases gradually with time and thus the spin-squeezing decreases. When initial state is the spin coherent state, all the uncertainty of the collective spin on the x-y plane are identical as shown in Fig. 3(b) and the spin-squeezing parameter 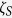 keeps constant. During the collective spontaneous emission, the upper-state population of the atomic ensemble 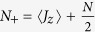 will decrease with time for both initial states, which are shown in Fig. 3(c,d). The initial population of the spin squeezed state [Fig. 3(c)] is larger than that of the spin coherent state [Fig. 3(d)]. The time evolution of the population of atomic upper-state is simulated by the evolution of photon numbers of the qu-mode.

### Fidelity of the quantum simulation system

We use fidelity 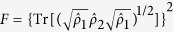, which denotes the overlap between the experimentally obtained output state 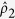 and the theoretically calculated final state 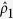 of the simulated QHO after the time evolution, to quantify the performance of the quantum simulation. The fidelity for two Gaussian states 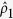 and 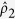 with the covariance matrices σ_*j*_ and the mean amplitudes 
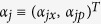
 (*j* = 1, 2) is expressed by^45^


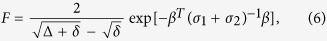


where 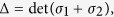 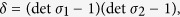 *β* = α_2_ − α_1_, σ_1_ and σ_2_ are the covariance matrices for the theoretically calculated 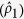 and experimentally obtained 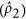 output states, respectively. In our system, if the ancillary entangled state with infinite squeezing (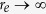) is utilized, the fidelity will equal to 1 under the ideal situation. When the entangled state (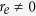) is used as the ancillary modes, the fidelity of the output state is higher than its classical limit, which is obtained by using the coherent states (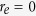) to replace the entangled states^21^,^25^,^26^. The experimentally measured fidelity is higher than the classical limit and thus the obtained results enter in the quantum regime. The covariance matrices are obtained from the noise power spectrum of the output state at 2 MHz, which is measured by HD3 and analyzed with a spectrum analyzer.

**Figure 4:**
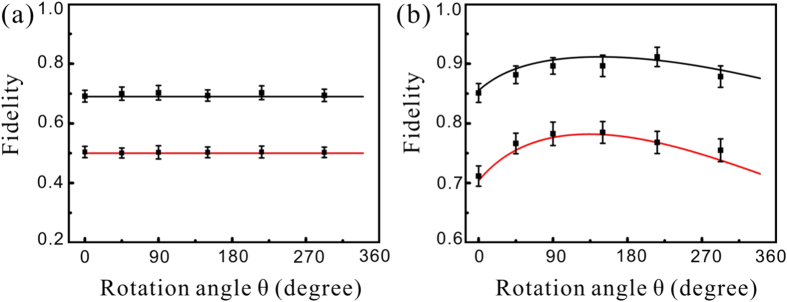
The fidelity of the quantum simulation. (**a**,**b**) Are the fidelity of the QHO with an input coherent state and amplitude-squeezed state in an open system, respectively. The bottom (red) and upper (black) lines stand for the classical limit and fidelities theoretically calculated with experimental parameters, respectively.

Figure 4 shows the fidelity of the QHO with an input coherent state [Fig. 4(a)] and squeezed state [Fig. 4(b)] in an open system, respectively. The bottom (red) and upper (black) lines stand for the classical limit and the theoretical fidelity with experimental parameters, respectively. The values calculated based on the experimentally measured results are marked by the black dots. The classical limit is obtained by replacing the EPR entangled state with a coherent state (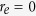) as the ancillary state in the CVQOS. It is obvious that the measured fidelity with EPR entangled state as the ancillary state is higher than the corresponding classical limit, which confirms the quantum feature of the QS system. When the attenuation exists, the fidelity of a squeezed state is not linearly decreased with the increase of the attenuation [Fig. 4(b)]. There is an optimal attenuation, where the best fidelity is reached and then it decreases. Finally the output state trends to a coherent state for large attenuations (In Fig. 4 the value of 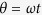 corresponds to the attenuation of 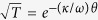).

## Discussion

For the conclusion, we have theoretically and experimentally demonstrated that the real-time evolution of the quantum state of a QHO can be simulated with a qu-mode and a logical operation in CVQC. The dynamics of an atomic ensemble in both spin coherent and spin squeezed states during the collective spontaneous emission are mimicked by the CVQOS. However, due to the restrictions in the use of HPT, the simulator is not able to fully simulate some complex dynamics of atomic systems, such as the superradiant emission of an ensemble where all atoms are initially excited. Besides, in this work the atomic interaction and some possible sources of decoherence are not contained. The environment noise we presented is only the vacuum noise. Under the influence of different environment noise, the evolution of the quantum state will be different. Thus the simulation system has to be totally renewed, that is out of the range of present manuscript. We hope that our work would attract the interests of theoretical and experimental physicists who can design more complete CVQOS.

The presented simulation system based on CVQC opens a new path to implement QS applying the mature quantum optical technologies. It may be used to simulate more complicated dynamical processes of quantum systems, whose Hamiltonian is described by the position and momentum operators. Generally, the Hamiltonian of the simulated system can be decomposed into a set of unitary transformations^46^, so its dynamic behavior will possibly be simulated by a sequence of quantum logic operations in CVQC and the coupling of qu-modes. For example, the simulation of interaction between two bosonic oscillators with strong coupling^32^ could be implemented by CVQOS. The Hamiltonian of two interacting bosonic oscillators is 
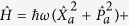

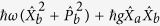
 (*g* is the interaction coefficient between two oscillators 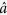 and 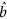), which can be decomposed into a sequence of quantum logic operations including rotation, squeezing and coupling on beam-splitters. A variety of CVQC operations have been experimentally demonstrated^24^,^25^,^26^, and the technologies used in these experiments can be directly utilized in a variety of CVQOS systems. We expect that the method can be extended to simulate more dynamic processes of QHO systems by applying multipartite entangled states and increasing the number of used qu-modes.

## Additional Information

**How to cite this article**: Deng, X. *et al.* Continuous variable quantum optical simulation for time evolution of quantum harmonic oscillators. *Sci. Rep.* **6**, 22914; doi: 10.1038/srep22914 (2016).

## Supplementary Material

Supplementary Information
