# Supplementary material for: Continuous variable quantum optical simulation for time evolution of quantum harmonic oscillators
Source: Sci Rep. 2016 Mar 10;6:22914. doi: 10.1038/srep22914 (PMC4785386; doi:10.1038/srep22914)
Supplement: Supplementary Information [file srep22914-s1.pdf]

Supplementary information for "Continuous variable quantum optical simulation for time evolution of quantum harmonic oscillators"

Xiaowei Deng,[1,2] Shuhong Hao,[1,2] Hong Guo,[1,3] Changde Xie[1,2] and Xiaolong Su[1,2*]

[1] *State Key Laboratory of Quantum Optics and Quantum Optics Devices, Institute of Opto-Electronics, Shanxi University, Taiyuan 030006, China*

[2] *Collaborative Innovation Center of Extreme Optics, Shanxi University, Taiyuan 030006, China*

[3] *College of Physical Science and Technology, Central China Normal University, Wuhan 430079, China*

[*] *Corresponding author, e-mail: suxl@sxu.edu.cn*

I. THE WIGNER FUNCTION OF THE OUTPUT STATE

The Wigner function of a qu-mode is

$$W(X,P)=\frac{\exp\{-\frac{1}{2}[(\mathbf{x}-\bar{\mathbf{x}})^T\,\sigma^{-1}\,(\mathbf{x}-\bar{\mathbf{x}})]\}}{2\pi\sqrt{\det\sigma}}, \tag{1}$$

where $\mathbf{x}=(X,P)^T$ and $\bar{\mathbf{x}}=(\ \langle X\rangle\ ,\ \langle P\rangle\ )^T$ represent the vector and the mean value (displacement) of the amplitude and phase quadratures respectively, and $\det\sigma$ is the determinant of covariance matrix σ. The covariance matrix of the input state is

$$\sigma_{in}=\frac{1}{4}\begin{bmatrix} V_{sq} & 0 \\ 0 & V_{an} \end{bmatrix}, \tag{2}$$

where V_{sq}, V_{an} are the variances of the squeezing and anti-squeezing component, respectively. The covariance matrix of the output state after the rotation operation in an open system ($T<1$) is expressed by

$$\sigma_{out}=\begin{bmatrix} V_X & V_{XP} \\ V_{XP} & V_P \end{bmatrix}, \tag{3}$$

where

$$V_X=\langle X^2\rangle-\langle X\rangle^2=V_{\min}\cos^2\theta+V_{\max}\sin^2\theta, \tag{4}$$

$$V_P = \left\langle P^2 \right\rangle - \left\langle P \right\rangle^2 = V_{\min} \sin^2\theta + V_{\max}\cos^2\theta, \quad (5)$$

$$V_{XP} = \frac{1}{2}\left(\left\langle XP \right\rangle + \left\langle PX \right\rangle\right) - \left\langle X \right\rangle \left\langle P \right\rangle = \left(V_{\max} - V_{\min}\right)\sin\theta\cos\theta, \quad (6)$$

$$V_{\min} = \frac{R[2e^{-2r_e} + T(V_{sq} - 1)] + 1}{4}, \quad (7)$$

and

$$V_{\max} = \frac{R[2e^{-2r_e} + T(V_{an} - 1)] + 1}{4}, \quad (8)$$

in which $R = 99\%$ is the reflection coefficient of the 99%R beam-splitter. When $V_{sq} = V_{an} = 1$, the covariance matrix of the output state is reduced to

$$\sigma_{out}^{C} = \frac{1}{4}\begin{bmatrix} 1 + 2Re^{-2r_e} & 0 \\ 0 & 1 + 2Re^{-2r_e} \end{bmatrix}, \quad (9)$$

which corresponds to the case that a coherent state is used as input state.

The average amplitudes of the input state are given by

$$\begin{bmatrix} \left\langle X \right\rangle_{in} \\ \left\langle P \right\rangle_{in} \end{bmatrix} = \begin{bmatrix} |\alpha|\cos\varphi_0 \\ |\alpha|\sin\varphi_0 \end{bmatrix}. \quad (10)$$

In the experiment, the average amplitudes of the input state are $\left\langle X \right\rangle_{in} = 1.58$ and $\left\langle P \right\rangle_{in} = 0$, where $\varphi_0 = 0$ has been chosen in Eq. (10) for simplicity. The average amplitudes of the output state are

$$\begin{bmatrix} \left\langle X \right\rangle \\ \left\langle P \right\rangle \end{bmatrix} = \sqrt{RT}\begin{bmatrix} \cos\theta\left\langle X \right\rangle_{in} + \sin\theta\left\langle P \right\rangle_{in} \\ \cos\theta\left\langle P \right\rangle_{in} - \sin\theta\left\langle X \right\rangle_{in} \end{bmatrix}. \quad (11)$$

II. TIME EVOLUTION OF AN ATOMIC ENSEMBLE

A spin coherent (squeezed) state of an atomic ensemble is mapped onto a coherent (squeezed) state of a QHO (qu-mode), respectively. The initial spin coherent state $\left|\frac{\alpha}{\sqrt{N}}\right\rangle_a$ ($|\alpha|^2 \ll N$) of an atomic ensemble can be mapped onto a coherent state $|\alpha\rangle$ of a qu-mode harmonic oscillator,

$$\left|\frac{\alpha}{\sqrt{N}}\right\rangle_a = \frac{1}{(1 + |\alpha|^2/N)^{N/2}}\sum_{n_a=0}^{N}\left(\frac{\alpha}{\sqrt{N}}\right)^{n_a}\sqrt{\frac{N!}{(N-n_a)!n_a!}}\left|\frac{N}{2}, n_a - \frac{N}{2}\right\rangle_a, \quad (12)$$

where $\left|\frac{N}{2}, n_a - \frac{N}{2}\right\rangle_a$ is the eigenvector of $\hat{J}^2$ and $\hat{J}_z$ with eigenvalue $\frac{N}{2}(\frac{N}{2}+1)$ and $m = n_a - N/2$, respectively, and

$$|\alpha\rangle = e^{-|\alpha|^2}\sum_{n=0}^{\infty}\frac{\alpha^n}{\sqrt{n!}}|n\rangle, \tag{13}$$

where $|n\rangle$ is the eigenvector of $\hat{a}^\dagger\hat{a}$ with eigenvalue n and $\alpha = |\alpha| e^{i\varphi_0}$. Substituting Eq. (2) with $V_{sq} = V_{an} = 1$ into Eq. (1), the corresponding Wigner function of the initial spin coherent state $\left|\frac{\alpha}{\sqrt{N}}\right\rangle_a$ is given by

$$W(X,P) = \frac{2}{\pi}\exp\{-2[(X - |\alpha|\cos\varphi_0)^2 + (P - |\alpha|\sin\varphi_0)^2]\}. \tag{14}$$

The initial spin squeezed state $\left|\frac{\beta}{\sqrt{N}}, \frac{\zeta}{N}\right\rangle_a$ of an atomic ensemble can be mapped onto a coherent squeezed state $\hat{S}(\zeta)\hat{D}(\beta)|0\rangle$ of a qu-mode harmonic oscillator,

$$\left|\frac{\beta}{\sqrt{N}}, \frac{\zeta}{N}\right\rangle_a = \exp(-\frac{\zeta}{2N}\hat{J}_+^2 + \frac{\zeta^*}{2N}\hat{J}_-^2)\left|\frac{\beta}{\sqrt{N}}\right\rangle_a \xrightarrow{N\gg 1} \exp(-\frac{\zeta}{2}\hat{a}^{\dagger 2} + \frac{\zeta^*}{2}\hat{a}^2)|\beta\rangle = \hat{S}(\zeta)\hat{D}(\beta)|0\rangle, \tag{15}$$

here $\hat{S}(\zeta) = \exp(-\frac{\zeta}{2}\hat{a}^{\dagger 2} + \frac{\zeta^*}{2}\hat{a}^2)$ is the squeezing operator with $\zeta = re^{i\varphi_s}$ ($V_{sq} = e^{-2r}$) and $D(\beta) = \exp(\beta\hat{a}^\dagger - \beta^*\hat{a})$ is the displacement operator. In quantum optics, we have $\hat{S}(\zeta)\hat{D}(\beta)|0\rangle = \hat{D}(\alpha)\hat{S}(\zeta)|0\rangle$, where $\beta = \alpha\cosh r + \alpha^* e^{i\varphi_s}\sinh r$. The Wigner function of $\hat{D}(\alpha)\hat{S}(\zeta)|0\rangle$ is

$$W(X,P)_s = \frac{2}{\pi}\exp\{-2[V_{an}(X - |\alpha|\cos\varphi_0)^2 + V_{sq}(P - |\alpha|\sin\varphi_0)^2]\}. \tag{16}$$

When $V_{sq} = \frac{1}{V_{an}}$, it stands for a pure squeezed state. If $V_{sq} = V_{an} = 1$, Eq. (16) is reduced to Eq. (14). Substituting Eq. (2) into Eq. (1), we obtain the corresponding Wigner function of the initial spin squeezed state, which takes the same form of Eq. (16) with $V_{sq} = 0.45, V_{an} = 7.08$ for the squeezed state in the experiment (corresponding to measured squeezing and anti-squeezing noises of the quadrature-amplitude squeezed state are -3.5 dB and 8.5 dB).

According to the average amplitudes of the output state in Eq. (11) and the relationship between the collective spin operators and the position (momentum) operators of a harmonic oscillator, the mean values of the collective spin operators equal to

$$\langle J_x\rangle = \sqrt{NR}\alpha e^{-\kappa t}\cos(\omega t), \tag{17}$$

$$\langle J_y\rangle = \sqrt{NR}\alpha e^{-\kappa t}\sin(\omega t), \tag{18}$$

$$\langle J_z \rangle = R(\alpha^2 + \frac{V_{sq} + V_{an}}{4} - \frac{1}{2})e^{-2\kappa t} + Re^{-2r_e} - \frac{N}{2}. \tag{19}$$

The total upper-state population of the atomic ensemble N_+ equals to

$$N_+ = \langle J_z \rangle + \frac{N}{2} = R(\alpha^2 + \frac{V_{sq} + V_{an}}{4} - \frac{1}{2})e^{-2\kappa t} + Re^{-2r_e}. \tag{20}$$

Obviously, in the case of the perfect EPR entangled state ($r_e \longrightarrow \infty$), the decay rate of the total upper-state population N_+ is 2κ, which is proportional to the total number N of atoms in the atomic ensemble because of $g_k = g_k^a \sqrt{N}$.

From Eqs. (17) - (19), the collective spin average vector is almost invariable in the negative direction of z axis due to $N \gg 1$. So we need only to consider the uncertainty in the component of the collective spin on the x-y plane. Based on the Wigner function of the output state in an open system and the relationship between the collective spin operators and the position (momentum) operators of a harmonic oscillator, we obtain

$$\triangle J_\perp = [(\triangle J_x)^2 \cos^2 \varphi + (\triangle J_y)^2 \sin^2 \varphi + Cov(J_x \cdot J_y) \sin(2\varphi)]^{1/2}, \tag{21}$$

where φ is the angle between $J_\perp$ and x axis, and

$$(\triangle J_x)^2 = NV_X, \tag{22}$$

$$(\triangle J_y)^2 = NV_P, \tag{23}$$

$$Cov(J_x \cdot J_y) = \frac{1}{2}(\langle J_x J_y \rangle + \langle J_y J_x \rangle) - \langle J_x \rangle \langle J_y \rangle = NV_{XP}. \tag{24}$$

By substituting Eqs. (22) - (24) and Eqs. (4) - (6) into Eq. (21), we have

$$\triangle J_\perp = \sqrt{N[V_{\min} \cos^2(\omega t - \varphi) + V_{\max} \sin^2(\omega t - \varphi)]}. \tag{25}$$

When $\omega t - \varphi = (2m+1)\pi$, uncertainty $\Delta J_\perp$ of the component $J_\perp$ of the collective spin is minimal, i.e. $\min(\Delta J_\perp) = \sqrt{NV_{\min}}$, when $\omega t - \varphi = (2m+1)\pi/2$, uncertainty $\Delta J_\perp$ of the component $J_\perp$ is maximal, $\max(\Delta J_\perp) = \sqrt{NV_{\max}}$.

Substituting experimental parameters into Eqs. (20) and (25), we obtain the dynamic behavior of the atomic ensemble in the collective spontaneous emission. During the collective spontaneous emission, the upper-state population of the atomic ensemble are calculated from Eq. (25).

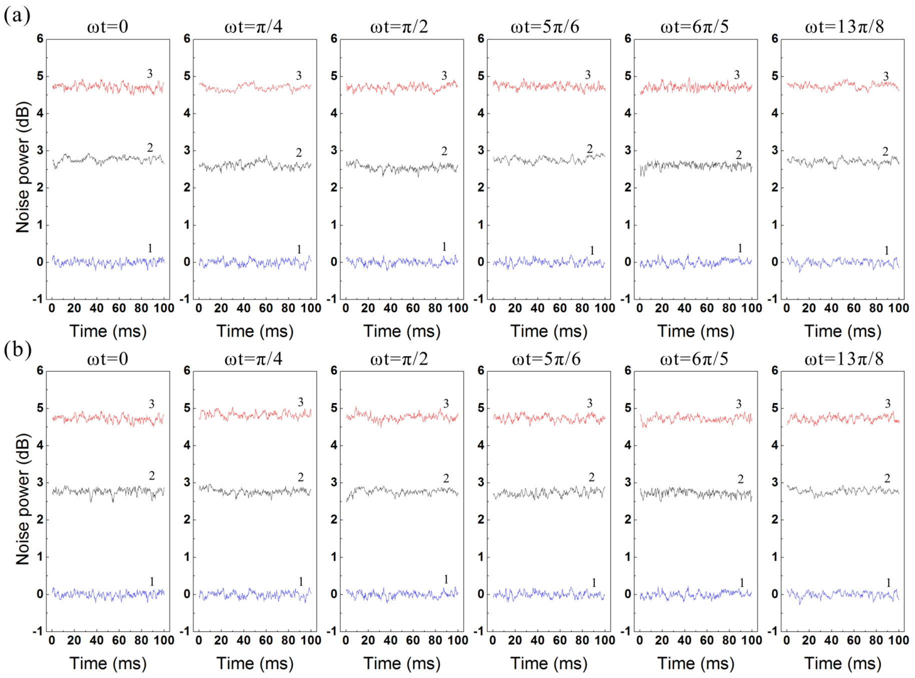

FIG. 1: Measured noise powers of the amplitude (a) and phase (b) quadratures of the output state with a coherent state as the input state in an open system for different rotation angles. The noise power of the output state is independent on the rotation angles. Trace 1 (blue line) is the SNL, traces 2 and 3 (black and red lines) are noise powers of the output states with and without EPR entangled state as the ancillary state, respectively. Measurement frequency is 2 MHz. The spectrum analyzer resolution bandwidth is 30 kHz and the video bandwidth is 100 Hz.

III. THE COVARIANCE MATRIX IN THE EXPRESSION OF FIDELITY

The covariance matrix σ_2 of the output mode is given by $\sigma_2 = 4\sigma_{out}$. The coefficient "4" comes from the normalization of shot noise level (SNL). Since the noise of a vacuum state is defined as 1/4, while in the fidelity formula the vacuum noise is normalized to "1", so a coefficient "4" appears in the expressions of covariance matrices. For the theoretically calculated final state $\hat{\rho}_1$ the covariance matrix is

$$\sigma_1 = 4 \begin{bmatrix} V_{X_1} & V_{XP_1} \\ V_{XP_1} & V_{P_1} \end{bmatrix} \tag{26}$$

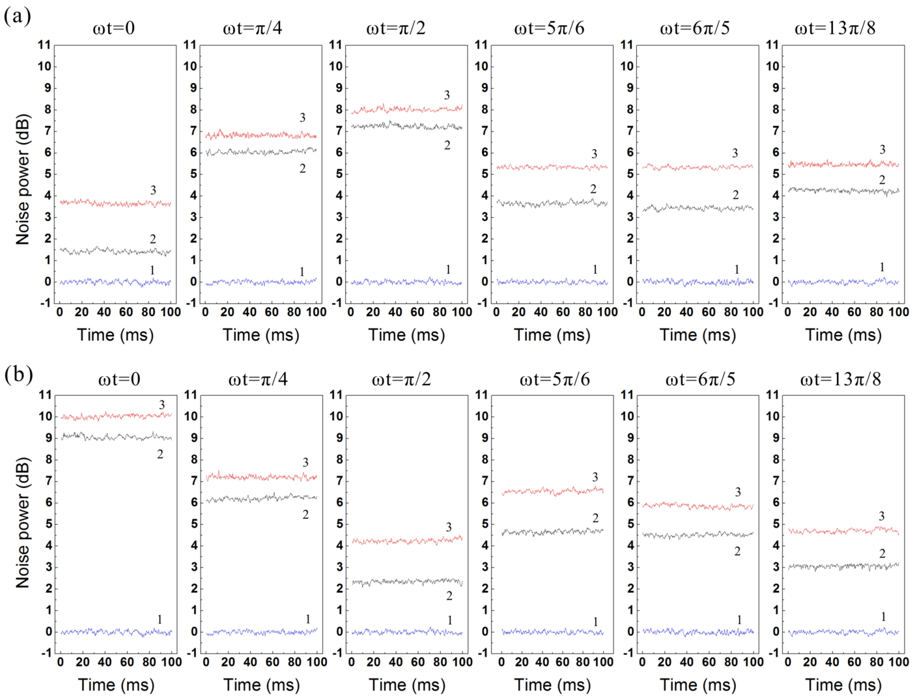

FIG. 2: Measured noise powers of the amplitude (a) and phase (b) quadratures of the output state with the input of an amplitude-squeezed state in an open system for different rotation angles. Trace 1 (blue line) is the SNL, traces 2 and 3 (black and red lines) are noise powers of the output state with and without EPR entanglement, respectively. Measurement frequency is 2 MHz. The spectrum analyzer resolution bandwidth is 30 kHz and the video bandwidth is 100 Hz.

where V_{X_1}, V_{P_1} and V_{XP_1} correspond to V_X, V_P and V_{XP} in Eq. (3) with $R = 1$ and $r_e \to \infty$, respectively. If $V_{sq} = V_{an} = 1$, the above equation becomes $\sigma_1 = \begin{bmatrix} 1 & 0 \\ 0 & 1 \end{bmatrix}$, which is the covariance matrix of a coherent state. The average amplitudes of the theoretically calculated final state $\hat{\rho}_1$ are obtained from Eq. (11) with $R = 1$. For the experimentally obtained output state $\hat{\rho}_2$, the elements in the covariance matrix is obtained from the experimentally measured noise powers of the output state.

The covariance matrices are obtained from the noise power spectrum of the output state at 2 MHz, which is measured by HD3 (shown in Fig. 1(b) in the main text) and analyzed with a spectrum analyzer. The measured noise powers of the output states with a coherent state and an amplitude-squeezed state as the input state at different rotation angles are shown in Fig. 1 and Fig. 2, respectively. During the measurement, the modulation signal

on EOM3 (shown in Fig. 1(b) in the main text) is removed. In Fig. 1, the measured noise power of the output state without EPR entanglement (trace 3, red line) is about 4.7 dB above the SNL (trace 1, blue line). The noise powers of the output state with EPR entanglement (trace 2, black line) are about 2.0 dB below than that without EPR entangled state at different rotation angles. The measured noise powers of the output state without and with EPR entanglement for an amplitude-squeezed state as input are shown in Fig. 2. It is obvious that the noise powers of the output state depend on the rotation angle because of the noise property of the input squeezed state.
